# Mindfulness-Based Interventions to Reduce Burnout in Primary Healthcare Professionals: A Systematic Review and Meta-Analysis

**DOI:** 10.3390/healthcare9101342

**Published:** 2021-10-09

**Authors:** Mafalda Salvado, Diogo Luís Marques, Ivan Miguel Pires, Nádia Mendes Silva

**Affiliations:** 1 Centro de Saúde Dr. Gorjão Henriques, Unidade de Saúde Familiar Cidade do Lis, 2410-272 Leiria, Portugal; nrsilva@arscentro.min-saude.pt; 2 Department of Sport Sciences, University of Beira Interior, 6201-001 Covilhã, Portugal; diogo.marques@ubi.pt; 3 Instituto de Telecomunicações, Universidade da Beira Interior, 6200-001 Covilhã, Portugal; impires@it.ubi.pt; 4 Escola de Ciências e Tecnologias, University of Trás-os-Montes e Alto Douro, Quinta de Prados, 5001-801 Vila Real, Portugal

**Keywords:** mindfulness, burnout, emotional exhaustion, depersonalization, personal accomplishment, primary healthcare professionals, meta-analysis

## Abstract

Mindfulness-based interventions (MBIs) are reported by experimental studies as practical approaches to reduce burnout in primary healthcare professionals (PHCP). However, to date, no research has synthesized the evidence to determine the overall effects of MBIs for reducing burnout in PHCP. We conducted a systematic review and meta-analysis to analyze the effects of MBIs to reduce burnout in PHCP. We searched articles in the PubMed/MEDLINE, Web of Science, Cochrane, and Scopus databases from inception to September 2021 using MeSH terms: “mindfulness”, “burnout”, and “primary healthcare”. Two reviewers extracted the data and assessed the risk of bias. We used a random-effects meta-analysis to calculate the standardized mean differences (SMD) and mean differences (MD) with 95% confidence intervals (CI) of emotional exhaustion (EE), depersonalization (DP), and personal accomplishment (PA) domains of burnout. Of 61 records, ten were included (*n* = 417). Overall, the studies were rated as having a high risk of bias and limited quality evidence. MBIs significantly reduced EE (SMD = −0.54, 95%CI: −0.72 to −0.36; MD = −5.89, 95%CI: −7.72 to −4.05), DP (SMD = −0.34, 95%CI: −0.52 to −0.17; MD = −1.96, 95%CI: −2.96 to −0.95), and significantly increased PA (SMD = 0.34, 95%CI: 0.17 to 0.52; MD = 2.05, 95%CI: 1.04 to 3.06). Although further high-quality research is needed, our findings support the implementation of MBIs for reducing burnout in PHCP.

## 1. Introduction

The modern healthcare system exposes professionals to various occupational stressors, including high workload, long working hours, time pressure, uncertainty regarding patient treatment, and human suffering [1,2,3]. This work-related stress may lead healthcare professionals to severe distress and burnout [1,2]. Consequently, these symptoms may negatively affect the quality of their service provision, the patient’s health and outcomes, and the viability of the healthcare system [1,2,4]. The economic burden of burnout was estimated to be approximately 4.6 billion dollars per year in the USA [5] and between 5 to 9 billion euros per year in European countries [6]. Therefore, it is evident that burnout represents a major occupational health problem among healthcare professionals and an enormous challenge for healthcare systems worldwide to overcome.

Burnout is a three-dimensional syndrome characterized by high emotional exhaustion, high depersonalization, and a diminished sense of personal accomplishment [7]. This symptom is highly prevalent among primary healthcare professionals (PHCP) [8,9,10,11,12]. In primary healthcare units, physicians and nurses are among the professionals who report the highest levels of burnout [10]. According to empirical evidence, primary care physicians are among the specialists who report the highest rates of burnout [12,13], and at least half will experience symptoms of burnout at some point during their careers [4,9,14]. Primary care nurses are also considered a burnout risk group [11]. In this sense, over the last ten years, the medical literature has proposed practical approaches to reduce burnout in PHCP, including yoga [3] and mindfulness-based interventions (MBIs) [8,9,15,16,17,18,19,20,21,22].

Mindfulness refers to a process of paying attention, on purpose, in the present moment, and nonjudgmentally [23,24,25]. The ultimate goal of MBIs is to cultivate mindfulness within individuals and incorporate its practice into daily life through contemplation meditation exercises, yoga, and other practices [24,26]. As a result, individuals naturally increase their ability to cope with adverse emotional events, generating a great sense of emotional balance and well-being [8,9]. Furthermore, this self-awareness in clinical practice reduces stress and burnout symptoms, and increases well-being and compassion among clinicians and patients [16,27,28]. According to empirical research, MBIs contribute to significantly reducing emotional exhaustion [8,9,15,16,17,18,21], depersonalization [8,9,16,17,20,21], and increase the sense of personal accomplishment in PHCP [8,9,15,16,17]. Moreover, MBIs also present a favorable effect on the physician-patient relationship, which is paramount in improving patients’ health outcomes [29].

Nevertheless, despite the promising results of MBIs for reducing burnout in PHCP, to the best of our knowledge, no systematic review and meta-analysis has determined the overall pooled effect of MBIs on burnout in PHCP. Combining the results from studies to obtain a more precise estimate of the effect of MBIs on burnout is essential for understanding the effectiveness and utility of MBIs in clinical practice. Therefore, the present systematic review and meta-analysis aimed to synthesize the scientific evidence and quantify the pooled effect of MBIs on the burnout syndrome in PHCP. Based on the previous experimental studies mentioned above, we hypothesized that the pooled effects of MBIs would reveal a significant improvement on different burnout symptoms in PHCP.

## 2. Materials and Methods

### 2.1. Search Strategy

This review followed the Preferred Reporting Items for Systematic Reviews and Meta-Analyses (PRISMA) guidelines [30]. We conducted a comprehensive search in the PubMed/MEDLINE, Web of Science, Cochrane Library, and Scopus web databases from inception to 1 September 2021 using a combination of MeSH terms: “mindfulness”, “burnout”, and “primary healthcare”. We excluded dissertations and theses, letters to the editor, reviews, meta-analyses, observational studies, studies published before 2000, and studies in languages other than English, Portuguese, and Spanish. We searched the references from the retrieved articles to find additional studies that met the inclusion criteria.

### 2.2. Eligibility Criteria

The inclusion criteria followed the PICOS approach [31]:

- Population (P): studies that included PHCP.
- Intervention (I): studies that analyzed the effects of MBIs on burnout in PHCP.
- Comparison (C): studies that presented the pre- and post-test results of the Maslach Burnout Inventory.
- Outcomes (O): studies that measured the emotional exhaustion, depersonalization, and personal accomplishment domains of the Maslach Burnout Inventory.
- Study design (S): randomized controlled trials (RCTs) and non-RCTs.

### 2.3. Study Selection and Data Extraction

We exported the results from the web databases to Microsoft Office Excel and removed the duplicates. Two reviewers (M.S. and D.L.M.) independently screened the titles and abstracts and reviewed the full texts. Disagreements were resolved by consensus. The data extracted consisted of the following: study (authors, date, country, design); population (sample size, sex, age, clinical practice experience); MBI characteristics (intervention duration, number of sessions per week, hours per session, and exercises); and outcomes (means and standard deviations or 95% confidence intervals (CI) of the Maslach Burnout Inventory domains). After an intervention, a decrease in burnout emotional exhaustion and depersonalization domains means a significant improvement, and an increase in personal accomplishment means a significant improvement [7]. When the included studies presented the results as mean with 95% CI, we converted the 95% CI to standard deviation (SD = √N × ((upper limit − lower limit)/3.92)) [32]. We calculated the mean percentage change (Δ = ((post-test − pre-test)/pre-test) × 100) in studies that presented the means for each outcome at pre- and post-test. In two studies, the authors used cut-off values to classify the participants in each domain of the Maslach Burnout Inventory and presented the results as N(%) for each domain category [18,19]. After email contact to request the data, the authors did not answer, and therefore we did not include these articles in the meta-analysis.

### 2.4. Study Quality and Strength of Recommendation

Two reviewers (M.S. and D.L.M.) independently assessed the quality of the included studies, and disagreements were resolved by consensus. For RCTs, we used the Cochrane Risk of Bias Tool 2.0 (RoB 2), which incorporates five domains: randomization process, deviations from intended interventions, missing outcome data, measurement of the outcome, and selection of the reported results [33]. The risk of bias judgment for each domain is interpreted as low risk, some concerns, or high risk [33]. For non-RCTs, we used the Risk of Bias in Non-Randomized Studies of Interventions (ROBINS-I), which evaluates seven domains of bias, classified by the time of occurrence: pre-intervention (confounding, selection of the study participants), at intervention (classification of intervention), and post-intervention (deviations from intended interventions, missing outcome data, measurement of the outcome, and selection of the reported results) [34]. The risk of bias judgment for each domain is interpreted as low risk, moderate risk, serious risk, critical risk, or no information [34]. To assess the level of evidence and strength of recommendation, we used the Strength of Recommendation Taxonomy (SORT) [35]. The SORT rates the level of evidence from 1 to 3 (level 1: good-quality patient-oriented evidence; level 2: limited-quality patient-oriented evidence; level 3: other evidence) and the strength of recommendation from A to C (A: recommendation based on consistent and good-quality patient-oriented evidence; B: recommendation based on inconsistent or limited quality patient-oriented evidence; C: recommendation based on consensus, usual practice, disease-oriented evidence, case series for studies of treatment or screening, and/or opinion) [35].

### 2.5. Statistical Analysis

We circumscribed the analysis to studies that used the 22-item Maslach Burnout Inventory. This scale is considered the gold standard tool to measure burnout in clinicians [13,14]. We used a random-effects model (DerSimonian–Laird approach) to determine the effects of MBIs on burnout by computing the standardized mean differences (SMD) with 95% CI between the pre- and post-test results of each Maslach Burnout Inventory domain (i.e., emotional exhaustion, depersonalization, and personal accomplishment). Along with the SMD results, we also reported the pooled mean difference to increase the clinical interpretability of the results [36]. We used the inverse variance method to weigh the studies. The magnitude of the SMD was interpreted as small (0.2–0.49), moderate (0.50–0.79), or large (≥0.80) [37]. The heterogeneity between studies was assessed using the inconsistency test (I^2^), where values above 25%, 50%, and 75% were interpreted as low, moderate, and high heterogeneity, respectively [38]. Statistical significance was set at *p*-value < 0.05. We conducted all analyses in the Review Manager software (RevMan v5.4, Cochrane Collaboration, Oxford, UK).

## 3. Results

### 3.1. Study Search Results

**Figure 1:**
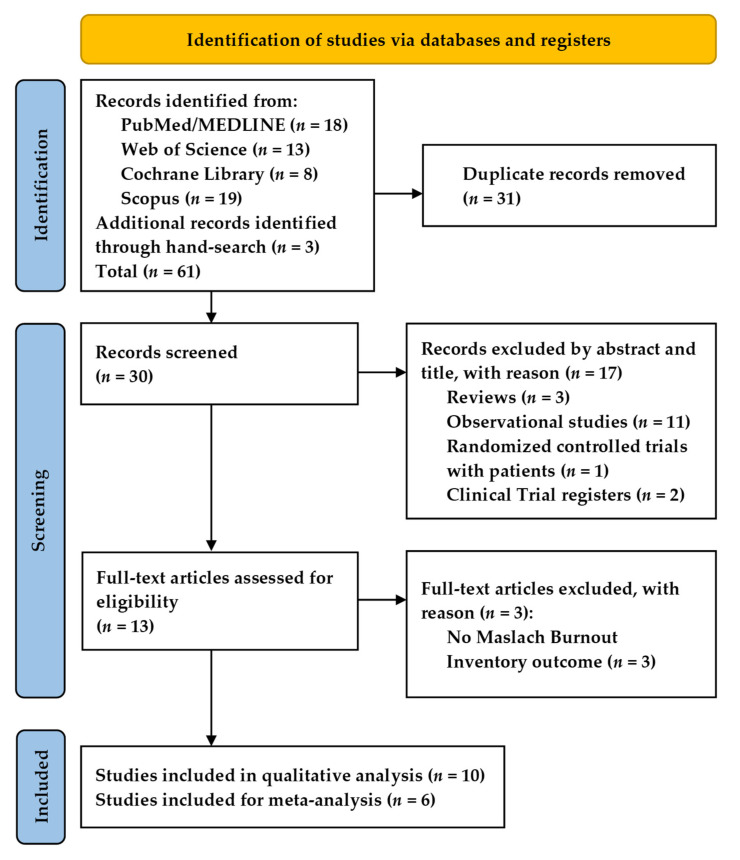
PRISMA flowchart for study inclusion.

The initial search yielded 61 records (Figure 1). After duplicate removal, we screened the titles and abstracts of 30 records, where 13 were eligible for full-text revision. We excluded three articles that did not use the Maslach Burnout Inventory to assess burnout. Therefore, ten studies met the inclusion criteria for the qualitative analysis. For the meta-analysis, we removed two studies due to the impossibility of extracting the data [18,19] and another two studies that did not use the 22-item Maslach Burnout Inventory [20,22]. Therefore, we included six studies for the meta-analysis.

### 3.2. Characteristics of the Included Studies

Table 1 presents the characteristics of the included studies. A total of 10 studies with 417 participants (46.6 ± 4.6 years old, 68% women) were included in this review. Four RCTs and six non-RCTs analyzed the impact of MBIs on burnout in PHCP. The proportion of physicians, nurses, and others (e.g., social workers and psychologists) was 78.5%, 20.1%, and 1.4%, respectively. The number of years in clinical practice was 18.3 ± 6.1 (range 9.8–24 years). In general, the participants reported no previous experience in mindfulness practices. The type of MBI course varied between studies. One study used the Continuing Medical Evaluation (CME) course [15], three studies used the Mindfulness-Based Stress Reduction (MBSR) course [8,17,20], two used a modified MBSR course [18,19], two used the Mindful Medicine Curriculum (MMC) course [9,16], and two used the modified Mindfulness-Based Cognitive Therapy (MBCT) course [21,22]. A full description of each course can be found in the Supplementary Materials (Table S1). The MBI courses ranged from 5 days to 8 weeks, the total hours from 16 to 28 h, and the duration of the sessions from 2 to 8 h. The MBI courses included mindfulness practices, such as speaking, listening, observing, contemplation meditation exercises, didactic exercises, dialogue groups, and yoga. The participants were also requested to practice mindfulness activities daily (range 10–45 min) during the courses. Regarding the outcome measures, eight studies used the 22-item Maslach Burnout Inventory [8,9,15,16,17,18,19,21], one study used the 20-item Maslach Burnout Inventory Dutch version [20], and one study used the 16-item Maslach Burnout Inventory Brazilian version [22]. After MBIs, the emotional exhaustion domain of burnout significantly decreased in seven studies (Δ = −22.1%, 95% CI, −28.2 to −16.0%) [8,9,15,16,17,18,21], while the depersonalization domain decreased in six studies (Δ = −21.1%, 95% CI, −25.3 to −16.9%) [8,9,16,17,20,21]. The sense of personal accomplishment significantly increased in five studies (Δ = 5.6%, 95% CI, 4.0 to 7.2%) [8,9,15,16,17].

### 3.3. Risk of Bias, Level of Evidence, and Strength of Recommendation Assessment

Table 2 presents the study quality and strength of recommendation for RCTs. According to RoB 2, the RCT studies presented an overall judgment of high risk of bias, which mainly arose from the bias in the measurement of the outcome domain. Regarding the SORT, the studies were rated as level 2 evidence, and the overall strength of recommendation was classified as B (limited-quality patient-oriented evidence).

Table 3 presents the study quality and strength of recommendation for non-RCTs. According to ROBINS-I, the non-RCT studies presented an overall judgment of serious risk of bias, which mainly arose from the bias in the measurement of the outcome domain. Regarding the SORT, the studies were rated as level 2 evidence, and the overall strength of recommendation was classified as B (limited-quality patient-oriented evidence).

### 3.4. Meta-Analysis Results

**Figure 2:**
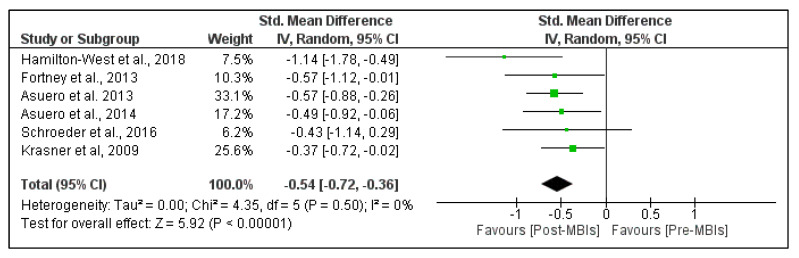
Effect of mindfulness-based interventions (MBIs) on emotional exhaustion in primary healthcare professionals. Results are reported as standardized mean difference and 95% confidence interval (CI).

Figure 2 shows the effects of MBIs on emotional exhaustion in PHCP. The pooled analysis revealed a moderate significant beneficial effect of MBIs on emotional exhaustion (SMD = −0.54; 95% CI, −0.72 to −0.36; *p*-value < 0.001). The heterogeneity was low, with I^2^ = 0%. The pooled mean difference was −5.89 points (95% CI, −7.72 to −4.05 points), which means a 5.89 point reduction in the emotional exhaustion domain of burnout (Figure S1).

**Figure 3:**
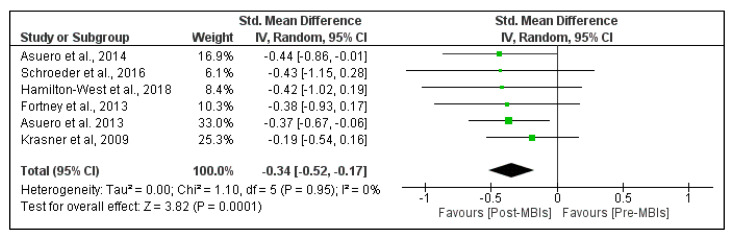
Effect of mindfulness-based interventions (MBIs) on depersonalization in primary healthcare professionals. Results are reported as standardized mean difference and 95% confidence interval (CI).

Figure 3 shows the effects of MBIs on depersonalization in PHCP. The pooled results found a small significant beneficial effect of MBIs on depersonalization (SMD = −0.34; 95% CI, −0.52 to −0.17; *p*-value < 0.001). The heterogeneity was low, with I^2^ = 0%. The pooled mean difference was −1.96 points (95% CI, −2.96 to −0.95 points), which means a 1.96 point reduction in the depersonalization domain of burnout (Figure S2).

**Figure 4:**
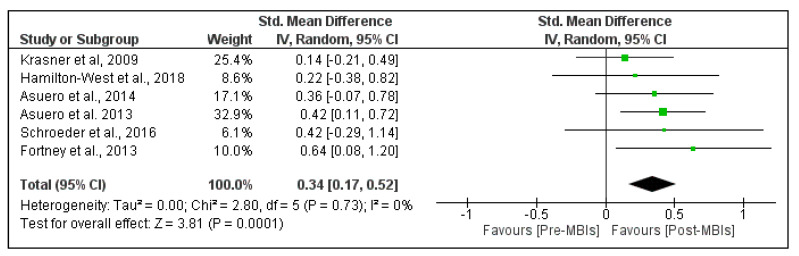
Effect of mindfulness-based interventions (MBIs) on personal accomplishment in primary healthcare professionals. Results are reported as standardized mean difference and 95% confidence interval (CI).

Figure 4 shows the effects of MBIs on personal accomplishment in PHCP. The pooled analysis demonstrated a small significant beneficial effect of MBIs on personal accomplishment (SMD = 0.34; 95% CI, 0.17 to 0.52; *p*-value < 0.001). The heterogeneity was low, with I^2^ = 0%. The pooled mean difference was 2.05 points (95% CI, 1.04 to 3.06 points), which means a 2.05 point increase in the personal accomplishment domain of burnout (Figure S3).

## 4. Discussion

### 4.1. Main Findings

To the best of our knowledge, this is the first systematic review and meta-analysis that aimed to synthesize and determine the overall effect of MBIs in reducing burnout in PHCP. The meta-analytical data suggest that MBIs effectively reduce burnout symptoms in PHCP, although with a small to moderate magnitude of effect. Nevertheless, despite the low heterogeneity between studies in the pooled analysis (I^2^ = 0%), these data should be interpreted cautiously due to the overall high risk of bias and limited-quality evidence observed in the included studies. Although previous reviews and meta-analyses also observed positive effects of MBIs in reducing burnout in healthcare professionals [1,4,39,40,41,42,43], the included studies were generally rated as low-quality evidence with a moderate to high risk of bias. The main reason for these ratings was the lack of blinding of participants and instructors to MBIs, which might have influenced the assessment of the outcome. Therefore, taken together, these results highlight the need for high-quality blinded RCTs to determine a more precise effect of MBIs on burnout in PHCP.

### 4.2. Effectiveness of MBIs on Emotional Exhaustion

Our findings substantiate that MBIs can moderately reduce emotional exhaustion in PHCP. The literature considers emotional exhaustion as one of the core components of burnout [44,45,46]. A state of extreme fatigue, inability to face the work demands, and psychologically support patients characterize clinicians’ emotional exhaustion [45,47]. A European study conducted on a large scale observed that 43% (95% CI, 41 to 46%) of family doctors (*n* = 599) reported high levels of emotional exhaustion [12]. In the same direction, a meta-analysis reported a prevalence of 28% (95% CI, 22 to 34%) of high emotional exhaustion in primary care nurses (*n* = 1110) [11]. Considering that high emotional exhaustion significantly predicts mortality among physicians and nurses [45], these results are concerning. Therefore, healthcare organizations should be encouraged to warn their professionals about the importance of preventing/reducing emotional exhaustion through evidence-based approaches, such as MBIs [41]. Previous meta-analyses that only analyzed the effects of MBIs found significant reductions of 2.6 points [41] and 4.68 points [4] on emotional exhaustion in physicians after interventions. Our pooled mean difference indicated a significant 5.89 point reduction in emotional exhaustion after MBIs. This result is higher than those observed in the previous meta-analyses, highlighting the effectiveness of MBIs in reducing burnout in PHCP. Considering that a 1 point increase in the emotional exhaustion domain of burnout is associated with a 6.9% increase in the odds of reporting suicidal ideation [48], a 6% increase in reporting medical errors [49], and a 43% increase in reduction in working hours [50], a decrease of 5.89 points should be considered a clinically meaningful change. Therefore, prescribing MBIs might be an effective strategy to prevent and reduce emotional exhaustion in PHCP. 

### 4.3. Effectiveness of MBIs on Depersonalization

The pooled analysis demonstrates that MBIs can produce small and significant improvements in the depersonalization domain of burnout in PHCP. Along with emotional exhaustion, depersonalization is considered the foundation of burnout [13,46]. Depersonalization describes a clinician’s detached feelings and impersonal treatment towards patients and negative attitudes towards colleagues [47,51,52]. An international cross-sectional study conducted on a large scale revealed that 35% (95% CI, 33 to 38%) of family doctors (*n* = 492) reported high levels of depersonalization [12]. In the same way, a meta-analysis showed that the prevalence of high depersonalization was 15% (95% CI, 9 to 23%) among primary care nurses (*n* = 1110) [11]. These results should alert PHCP of the importance of preventing/reducing depersonalization through effective approaches, such as MBIs. A previous meta-analysis with physicians that analyzed the pooled effects of MBIs observed a significant reduction of 2.01 points after the interventions [4]. Our pooled mean difference revealed a similarly small and significant reduction of 1.96 points in depersonalization after MBIs in PHCP. Considering that a 1 point increase in depersonalization is related to a 10.9% increase in the likelihood of reporting suicidal thoughts [48] and an 11% increase in committing medical errors [53], a decrease of 1.96 points should be considered a clinically meaningful change. Therefore, our meta-analytical findings reinforce the evidence regarding the importance and effectiveness of MBIs for reducing the depersonalization domain of burnout in PHCP.

### 4.4. Effectiveness of MBIs on Personal Accomplishment

Our meta-analytical data revealed a small and significant improvement in personal accomplishment after MBIs in PHCP. Reduced personal accomplishment is associated with a sense of not being capable of helping patients and effective at work [45,46]. A European study observed that 32% (95% CI, 30 to 35%) of family doctors (*n* = 445) reported low levels of personal accomplishment [12]. Similarly, a meta-analysis observed a prevalence of 31% (95% CI, 6 to 66%) of primary care nurses (*n* = 1110) reporting low personal accomplishment [11]. The sense of not being able to help others effectively establishes a significant correlation with clinician-rated patient safety [45]. This association means that the lower the personal accomplishment, the lower the clinician-rated patient safety, and vice versa [45]. Therefore, healthcare professionals reporting low levels of personal accomplishment must be supported with personal care interventions, which might include MBIs. According to our results, the pooled mean difference showed a small and significant increase of 2.05 points in personal accomplishment after MBIs in PHCP. To the best of our knowledge, this is the first reported result in the literature that exclusively reflects the effects of MBIs without pooling other interventions on personal accomplishment in healthcare professionals. A previous meta-analysis that analyzed the combined effects of MBIs with other interventions (e.g., organization direct interventions) in physicians, found a significant increase of 3 points in personal accomplishment after interventions. Taken together, these results substantiate the positive effects of MBIs for increasing the sense of personal accomplishment in healthcare professionals. Given that a 1 point decrease in the personal accomplishment domain of burnout is associated with a 5.7% increase in the odds of reporting suicidal ideation [48] and a 6% increase in reporting medical errors [49], an increase of 2.05 points should be considered a clinically meaningful change. Therefore, in light of these findings, MBIs may have special relevance to prevent and increase the diminished sense of personal accomplishment in PHCP. 

### 4.5. Strengths and Limitations

One strength of this research is that, to our best knowledge, it is the first comprehensive systematic review and meta-analysis to determine the pooled effect of MBIs for reducing burnout domains in PHCP. Contrary to previous meta-analyses that included physicians of all specialties [4,41], other healthcare professionals [39], or mixed interventions [42], we circumscribed our analysis only to MBIs and PHCP. Therefore, these results are only applicable to PHCP, which allows making specific inferences about the effectiveness of MBIs on a sector highly vulnerable to burnout [8,9,10,11,12]. Another strength of our review is related to the methodological design. Two independent reviewers searched and screened the data and assessed the quality of the studies, which helped to minimize biases and human error, and improve the validity and reliability of our review [54]. We also expanded the literature search to studies published in three languages. This comprehensive search resulted in the inclusion of two studies in the Spanish language. In the meta-analysis, the heterogeneity was low because we only included those that used the 22-item Maslach Burnout Inventory in the pooled analysis. Finally, along with the SMD, we also reported the pooled mean difference, which will allow clinicians and researchers to generalize the magnitude of the effect of MBIs on burnout in PHCP and increase the clinical interpretability of the results [36].

This study has, however, some limitations that we need to address. Firstly, the small number of included studies does not allow us to generalize the results, and therefore they should be considered preliminary. Moreover, publication bias, a threat to a meta-analysis’s validity, was not assessed because when there are fewer than 10 studies, the power is low to distinguish chance from real asymmetry [55]. Secondly, all included studies were associated with a high risk of bias in measuring the outcome domain by not blinding the participants, instructors, or assessors. Furthermore, according to SORT, the overall evidence and strength of recommendation were rated as limited-quality patient-oriented evidence. Therefore, future high-quality RCTs are needed to address the risk of bias highlighted in the included studies and increase the strength of recommendation of MBIs for reducing burnout in PHCP. Regardless of the above limitations, this systematic review and meta-analysis contribute to increasing the current scientific evidence about the impact of MBIs for reducing burnout symptoms in PHCP.

### 4.6. Practical Implications

The primary purpose of MBIs is to increase self-awareness in PHCP and help them cope with negative and stressful emotions during professional work [8,9,15,16,17,18,19,20,21,22]. Regular mindfulness practice among PHCP cultivates clear thinking and compassion for themselves and others, and generates a great sense of emotional balance and well-being, which is paramount for increasing the quality of care provided [8,9,15,16,17,18,19,20,21,22]. Furthermore, considering that emotional exhaustion (i.e., the core dimension of burnout) is associated with suicidal ideation among primary care physicians [56,57], cultivating mindfulness skills might help prevent and reduce suicidal thoughts in these medical specialists. Other expected benefits of MBIs in PHCP encompass improved mood, emotional stability, better self-care, reduced stress, empathy to the patients and colleagues, and a high sense of professionalism [8,9,15,16,17,18,19,20,21,22].

In this way, healthcare organizations should be encouraged to implement MBIs for PHCP ranging from 5 days to 8 weeks, with one weekly session of 2 to 2.5 h [8,9,15,16,17,18,19,20,21,22]. For PHCP who cannot regularly attend the sessions due to time constraints, a brief face-to-face MBI course of 5 days [9,16] or an online MBI course [40] is advisable to prevent dropouts. The mindfulness practices should include the core clinical skills of speaking, listening, and observing, as well as contemplation meditation exercises, didactic exercises, dialogue groups, and yoga. Furthermore, the participants should practice at least 10 min of mindfulness activities daily. Importantly, MBIs should guarantee a long-term improvement in well-being and professional accomplishment, and not only a short-term reduction in burnout. Therefore, considering the high rates of burnout among medical students and medical residents [58,59,60], implementing MBIs into medical school curricula might be an effective strategy to acquire mindfulness skills early and incorporate them throughout the career to prevent burnout [61].

## 5. Conclusions

The findings of this systematic review and meta-analysis suggest that MBIs might be a practical approach for inducing significant improvements in different burnout symptoms in PHCP. These findings can potentially be relevant for PHCP, mainly due to the exponential increase in reported cases of PHCP with burnout, recently exacerbated due to the COVID-19 pandemic [62]. Thus, from a practical standpoint, implementing MBIs in PHCP will reduce burnout and enhance well-being, compassion for themselves, colleagues, and patients, and contribute to sustainable healthcare organizations.

## Figures and Tables

**Table 1:** Characteristics of the included studies.

Study, Year, Country	Design	Sample	Mindfulness-Based Intervention	Outcomes	Results (Δ; *p*-Value)
Krasner et al. [15], 2009, USA	Non-RCT	70 PHCP (46% women; 100% physicians)	CME course of 8 weeks (27 h): 1 weekly session of 2.5 h, plus 1 extra session of 7 h; mindfulness meditation, didactic and narrative exercises, dialogue groups.	22-item Maslach Burnout Inventory (EE, D, PA)	EE: −14.7%; <0.01 D: −11.6%; >0.05 PA: 1.9%; <0.01
Asuero et al. [17], 2013, Spain	Non-RCT	87 PHCP (90% women; 55% physicians; 39% nurses; 6% social workers and psychologists); 47.3 ± 8.2 years	MBSR course of 8 weeks (28 h): 1 weekly session of 2.5 h, plus 1 extra session of 8 h; contemplation meditation exercises, dialogue groups.	22-item Maslach Burnout Inventory (EE, D, PA)	EE: −24.1%; <0.001 D: −23.3%; <0.001 PA: 6.5%; <0.001
Fortney et al. [9], 2013, USA	Non-RCT	30 PHCP (60% women; 97% physicians; 3% nurses); 40.5 ± 10.1 years	MMC course of 3 sessions (18 h): Friday (3 h) + Saturday (7 h) + Sunday (4 h) + 2 extra sessions of 2 h after the 2 and 4 weeks of the last sessions; mindfulness practices (sitting, movement, breathing, speaking, listening, observing, compassion).	22-item Maslach Burnout Inventory (EE, D, PA)	EE: −17.2%; <0.01 D: −18.3%; <0.05 PA: 8.1%; <0.001
Asuero et al. [8], 2014, Spain	RCT	43 PHCP (53% physicians; 40% nurses; 7% other); 48.8 ± 7.8 years	MBSR course of 8 weeks (28 h): 1 weekly session of 2.5 h, plus 1 extra session of 8 h; contemplation meditation exercises, dialogue groups.	22-item Maslach Burnout Inventory (EE, D, PA)	EE: −22.3%; <0.01 D: −26.5%; <0.01 PA: 5.4%; <0.01
Schroeder et al. [16], 2016, USA	RCT	16 PHCP (73% women; 100% physicians); 42.8 ± 8.4 years	MMC course of 3 sessions (18 h): Friday (3 h) + Saturday (7 h) + Sunday (4 h) + 2 extra sessions of 2 h after the 2 and 4 weeks of the last sessions; mindfulness practices (sitting, movement, breathing, speaking, listening, observing, compassion).	22-item Maslach Burnout Inventory (EE, D, PA)	EE: −15.8%; <0.001 D: −19.5%; <0.001 PA: 6.2%; <0.001
Verweij et al. [20], 2016, Netherlands	Non-RCT	23 PHCP (30% women; 100% physicians); 54.5 ± 5.3 years	MBSR course of 8 weeks (28 h): 1 weekly session of 2.5 h, plus 1 extra session of 8 h; contemplation meditation exercises, dialogue groups.	20-item Maslach Burnout Inventory Dutch version (EE, D, PA);	EE: −4.6%; >0.05 D: −11.2%; <0.05 PA: 3.4%; >0.05
Auserón et al. [18], 2017, Spain	RCT	23 PHCP (74% women; 52% physicians; 48% nurses); 50.0 ± 7.9 years	Modified MBSR course of 8 weeks (20 h): 1 weekly session of 2.5 h; contemplation meditation exercises, dialogue groups.	22-item Maslach Burnout Inventory (EE, D, PA)	EE: NA; <0.05 D: NA; >0.05 PA: NA; >0.05
Hamilton-West et al. [21], 2018, UK	Non-RCT	22 PHCP (64% women; 100% physicians); 44.5 ± 7.4 years	Modified MBCT course of 8 weeks (16 h): 1 weekly session of 2 h; contemplation meditation exercises, mindfulness practices, yoga.	22-item Maslach Burnout Inventory (EE, D, PA)	EE: −38.4%; <0.001 D: −27.7%; <0.05 PA: 5.6%; >0.05
Fuertes et al. [19], 2019, Spain	RCT	41 PHCP (83% women; 49% physicians; 51% nurses); 49.6 ± 8.2 years	Modified MBSR course of 8 weeks (20 h): 1 weekly session of 2.5 h; contemplation meditation exercises, dialogue groups.	22-item Maslach Burnout Inventory (EE, D, PA)	EE: NA; >0.05 D: NA; >0.05 PA: NA; >0.05
Sopezki et al. [22], 2020, Brazil	Non-RCT	62 PHCP (95% women); 41.7 ± 11.7 years	Modified MBCT course of 8 weeks (16 h): 1 weekly session of 2 h; mindfulness practices (breathing, speaking, listening, observing, compassion).	16-item Maslach Burnout Inventory Brazilian version (EE, D, PA)	EE: −9.6%; >0.05 D: −22.4%; >0.05 PA: 0.4%; >0.05

Δ, mean percentage change (%). CME, Continuing Medical Evaluation. EE, emotional exhaustion. D, depersonalization. MBSR, Mindfulness-Based Stress Reduction. MMC, Mindful Medicine Curriculum. MBCT, Mindfulness-Based Cognitive Therapy. NA, not applicable. PA, personal accomplishment. PHCP, primary healthcare professionals. RCT, randomized controlled trial.

**Table 2:** Risk of bias, level of evidence, and strength of recommendation for randomized controlled trials.

	Cochrane Risk of Bias Tool 2.0						SORT	
Study, Year	Randomization Process	Deviations from Intended Interventions	Missing Data	Measurement of Outcome	Selection of Reported Results	Overall Risk of Bias	Level of Evidence	Strength of Recommendation
Asuero et al. [8], 2014	Low risk	Some concerns	Low risk	High risk	Low risk	High risk	2	B
Schroeder et al. [16], 2016	Low risk	Some concerns	Low risk	High risk	Low risk	High risk	2	B
Auserón et al. [18], 2017	Low risk	Some concerns	Low risk	High risk	Low risk	High risk	2	B
Fuertes et al. [19], 2019	Low risk	Some concerns	Low risk	High risk	Low risk	High risk	2	B

SORT, strength of recommendation taxonomy.

**Table 3:** Risk of bias, level of evidence, and strength of recommendation for non-randomized controlled trials.

	Risk of Bias in Non-Randomized Studies of Interventions								SORT	
Study, Year	Confounding	Selection of Participants	Classification of Intervention	Deviations from Intended Interventions	Missing Data	Measurement of Outcome	Selection of Reported Result	Overall Risk of Bias	Level of Evidence	Strength of Recommendation
Krasner et al. [15], 2009	Moderate risk	Low risk	Low risk	Moderate risk	Low risk	Serious risk	Low risk	Serious Risk	2	B
Asuero et al. [17], 2013	Moderate risk	Low risk	Low risk	Moderate risk	Moderate risk	Serious risk	Low risk	Serious Risk	2	B
Fortney et al. [9], 2013	Moderate risk	Low risk	Low risk	Moderate risk	Low risk	Serious risk	Low risk	Serious Risk	2	B
Verweij et al. [20], 2016	Moderate risk	Low risk	Low risk	Moderate risk	Low risk	Serious risk	Low risk	Serious Risk	2	B
Hamilton-West et al. [21], 2018	Moderate risk	Low risk	Low risk	Moderate risk	Moderate risk	Serious risk	Low risk	Serious Risk	2	B
Sopezki et al. [22], 2020	Moderate risk	Low risk	Low risk	Moderate risk	Low risk	Serious risk	Low risk	Serious Risk	2	B

SORT, strength of recommendation taxonomy.

## Data Availability

Not applicable.

## Supplementary Materials

The following are available online at https://www.mdpi.com/article/10.3390/healthcare9101342/s1, Figure S1: Effect of mindfulness-based interventions (MBIs) on emotional exhaustion in primary healthcare professionals. Results are reported as mean difference and 95% confidence interval (CI); Figure S2: Effect of mindfulness-based interventions (MBIs) on depersonalization in primary healthcare professionals. Results are reported as mean difference and 95% confidence interval (CI); Figure S3: Effect of mindfulness-based interventions (MBIs) on personal accomplishment in primary healthcare professionals. Results are reported as mean difference and 95% confidence interval (CI); Table S1: Mindfulness-based intervention courses description.
