# Supplementary material for: Mindfulness-Based Interventions to Reduce Burnout in Primary Healthcare Professionals: A Systematic Review and Meta-Analysis
Source: Healthcare (Basel). 2021 Oct 9;9(10):1342. doi: 10.3390/healthcare9101342 (PMC8544467; doi:10.3390/healthcare9101342)
Supplement: Supplementary file 1 [file healthcare-09-01342-s001.zip › healthcare-1398067-supplementary.pdf]

Supplementary Materials

Mindfulness-based interventions to reduce burnout in primary healthcare professionals: a systematic review and meta-analysis

Figure S1: Effect of Mindfulness-Based Interventions (MBIs) on emotional exhaustion in primary healthcare professionals. Results are reported as mean difference and 95% confidence interval (CI).

Figure S2: Effect of Mindfulness-Based Interventions (MBIs) on depersonalization in primary healthcare professionals. Results are reported as mean difference and 95% confidence interval (CI).

Figure S3: Effect of Mindfulness-Based Interventions (MBIs) on personal accomplishment in primary healthcare professionals. Results are reported as mean difference and 95% confidence interval (CI).

Table S1. Mindfulness-based intervention courses description.

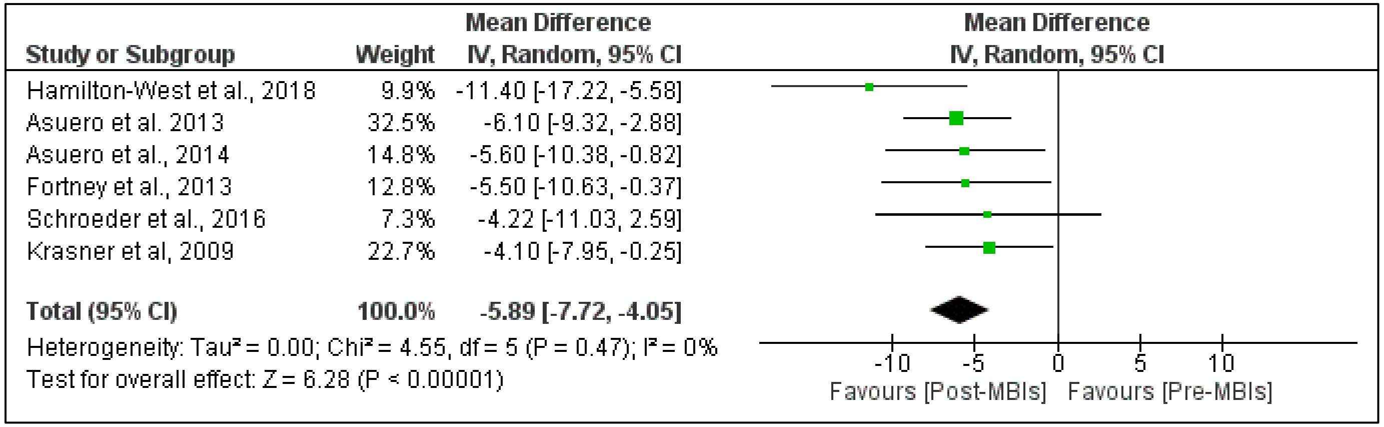

Figure S1: Effect of Mindfulness-Based Interventions (MBIs) on emotional exhaustion in primary healthcare professionals. Results are reported as mean difference and 95% confidence interval (CI).

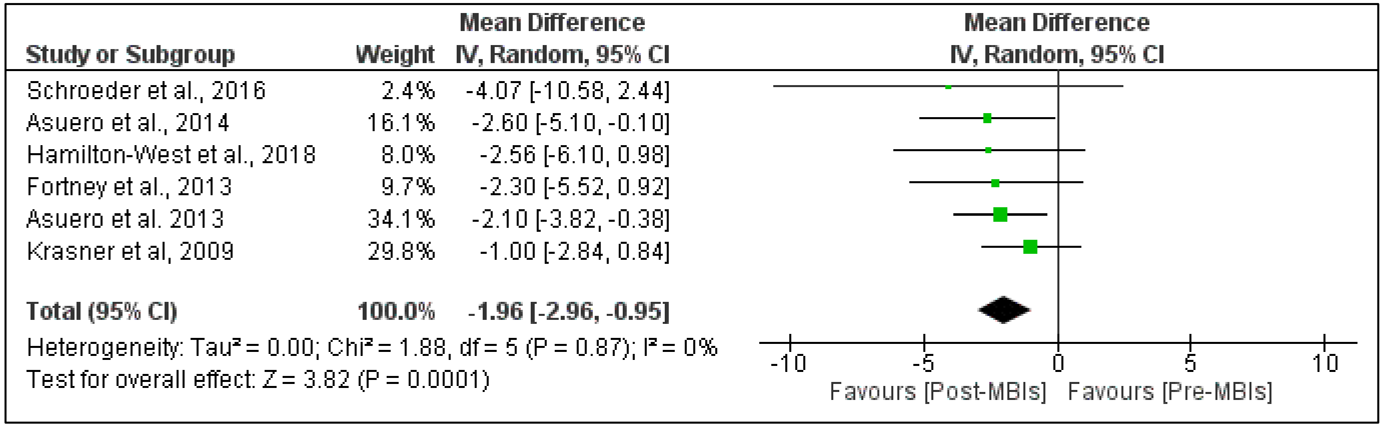

Figure S2: Effect of Mindfulness-Based Interventions (MBIs) on depersonalization in primary healthcare professionals. Results are reported as mean difference and 95% confidence interval (CI).

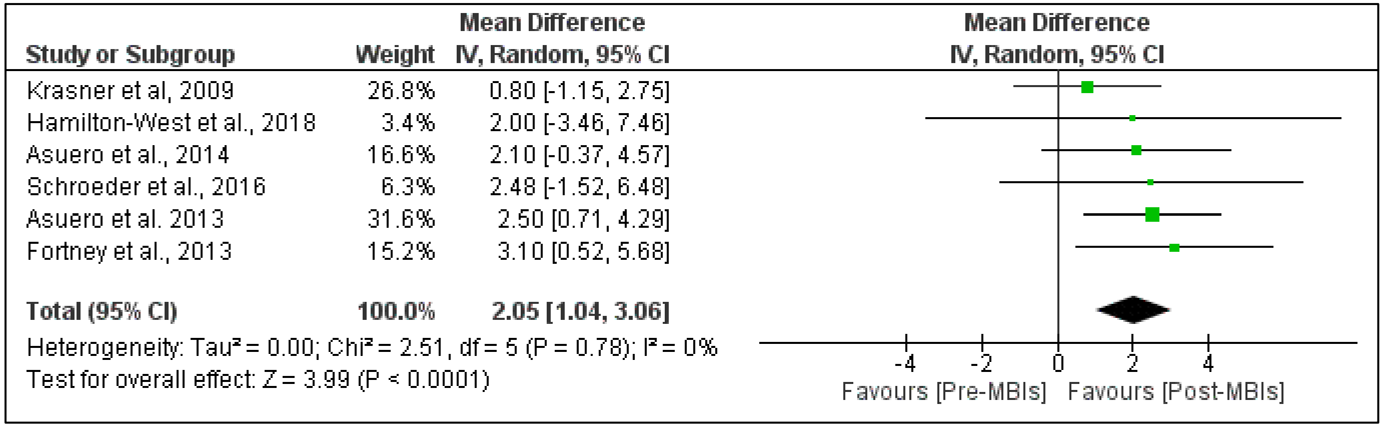

Figure S3: Effect of Mindfulness-Based Interventions (MBIs) on personal accomplishment in primary healthcare professionals. Results are reported as mean difference and 95% confidence interval (CI).

Table S1. Mindfulness-based intervention courses description.

Study, Year, Country	Course	Description
Krasner et al., 2009, USA	Mindfulness-Based Stress Reduction	• Course duration: 8 weeks plus an extra session of 7 h<br>• Session's duration: 1 weekly session of 2.5 h<br>• Content: Each weekly session comprised 4 training components: 1) didactic material (Topics included awareness of thoughts and feelings, perceptual biases and filters, dealing with pleasant and unpleasant events, managing conflict, preventing burnout, reflecting on meaningful experiences in practice, setting boundaries, examining attraction to patients, exploring self-care, being with suffering, and examining end-of-life care); 2) formal mindfulness meditation (body scan, sitting meditation, walking meditation, mindful movement); 3) narrative and appreciative inquiry exercises (sharing stories and experiences in small groups, and listening others); 4) discussion (large groups for discussing and sharing experiences of the formal mindfulness meditation practices and the narrative-appreciative inquiry exchanges)<br>• Instructor(s): Experienced instructors.
Asuero et al., 2013, Spain	Mindfulness-Based Stress Reduction	• Course duration: 8 weeks plus an extra session of 8 h<br>• Session's duration: 1 weekly session of 2.5 h

		• Content: session 1 – full consciousness, body scan, breath awareness; session 2 – breath awareness; session 3 – burnout, sitting yoga and breath awareness; session 4 – meaning in medicine, sitting yoga, breath awareness, and walking consciousness; session 5 – stress coping, stand-up yoga, and open consciousness meditation; session 6 – interpersonal attraction, open consciousness meditation; session 7 – self-care, open consciousness meditation; session 8 – suffering and end of life, body scan, open consciousness meditation; extra session – body scan and breath awareness, silent retreat, sitting and stand-up yoga, full consciousness walking, guided meditation, walking meditation, love and compassion meditation.<br>• Instructor(s): 1 certified Mindfulness-Based Stress Reduction (MBSR) instructor by the University of Massachusetts.
Fortney et al., 2013, USA	Mindful Medicine Curriculum	• Course duration: weekend (3 days) plus 2 follow-up sessions of 2 h<br>• Session's duration: 3 h on Friday evening, 7 h on Saturday, and 4 h on Sunday<br>• Content: Training in mindfulness practices (sitting, movement, speaking, listening, and compassion for self and others) and their application to practicing medicine and everyday life; 10-20 minutes daily mindfulness practice.<br>• Instructor(s): 2 certified MBSR instructors by the University of Massachusetts.
Asuero et al., 2014, Spain	Mindfulness-Based Stress Reduction	• Course duration: 8 weeks plus an extra session of 8 h<br>• Session's duration: 1 weekly session of 2.5 h

		• Content: contemplation-meditation exercises (focus on the present-moment experience and contemplate nonjudgmentally bodily sensations, breathing, sounds, and thoughts); each weekly session comprised four activities: 1) educational presentation (self-awareness, conflict management, burnout prevention, self-care, suffering, and end of life examination); 2) formal mindfulness meditation (notice, observe, and experience bodily sensations, thoughts, and feelings, acting with awareness and attention, focusing on experience without judgments); 3) narrative and appreciative inquiry exercises (writing and sharing personal experiences and intentionally listening others); 4) discussion (sharing experiences in group and discuss the effects of mindfulness practice).<br>• Instructor(s): 1 certified MBSR instructor.
Schroeder et al., 2016, USA	Mindful Medicine Curriculum	• Course duration: weekend (3 days) plus 2 follow-up sessions of 2 h<br>• Session's duration: 3 h on Friday evening, 7 h on Saturday, and 4 h on Sunday<br>• Content: Training in mindfulness practices (sitting, movement, speaking, listening, and compassion for self and others) and their application to practicing medicine and everyday life; 10-20 minutes daily mindfulness practice.<br>• Instructor(s): 1 instructor with extensive experience in mindfulness-based interventions.

Verweij et al., 2016, Netherlands	Mindfulness-Based Stress Reduction	• Course duration: 8 weeks plus an extra session of 8 h<br>• Session's duration: 1 weekly session of 2.5 h<br>• Content: sessions 1 and 2 – mindful movement and eating, body scan, breathing awareness, pleasant events calendar; sessions 3 and 4 – walking meditation, lying down mindful movement, unpleasant events calendar, stress response, standing mindful movement, group exploration of stress; sessions 5 and 6 – sitting and standing meditations, working with difficulties, responding instead of reacting, awareness meditation, mindful communications discussion; sessions 7 and 8 – silent meditations, loving-kindness meditation, formal and informal practice.<br>• Instructor(s): instructors experienced in MBSR.
Auserón et al., 2017, Spain	Modified Mindfulness-Based Stress Reduction	• Course duration: 8 weeks<br>• Session's duration: 1 weekly session of 2.5 h<br>• Content: session 1 – full consciousness, body scan; session 2 – perceptions and reality, meditation posture, mindfulness breathing; session 3 – emotions, pause for auto compassion, body scan, and yoga exercises; session 4 – burnout, walking mindfulness, body scan, and yoga exercises; session 5 – interpersonal relations, breathing mindfulness; session 6 – meaning in medicine, body scan, breathing awareness, stretching and body exercises with full consciousness; session 7 – time management, formal practices of mindfulness and compassion; session 8 – personal self-care plan, open consciousness meditation.

		• Instructor(s): 1 instructor experienced in MBSR.
Hamilton-West et al., 2018, UK	Modified Mindfulness-Based Cognitive Therapy	• Course duration: 8 weeks<br>• Session's duration: 1 weekly session of 2 h<br>• Content: session 1 – being aware of each moment, rather than being on automatic pilot; session 2 – focusing on the body and shifting away from the controlling chatter of the mind; session 3 – bringing attention to the breath to help achieve focus; session 4 – relating to experience in new ways; session 5 – accepting experience without judgment; session 6 – learning that thoughts are not facts; session 7 – identifying early warning signs and planning responses; session 8 – maintaining skills and taking care of self through mindfulness practice. The homework was less time-consuming than the original course and used the "Mindfulness: A Practical Guide to Finding Peace in a Frantic World" book as the homework text.<br>• Instructor(s): 1 instructor experienced in Mindfulness-Based Cognitive Therapy.
Fuertes et al., 2017, Spain	Modified Mindfulness-Based Stress Reduction	• Course duration: 8 weeks<br>• Session's duration: 1 weekly session of 2.5 h<br>• Content: session 1 – full consciousness, body scan; session 2 – perceptions and reality, meditation posture, mindfulness breathing; session 3 – emotions, pause for auto compassion, body scan, and yoga exercises; session 4 – burnout, walking mindfulness, body scan, and yoga exercises; session 5 – interpersonal relations,

		breathing mindfulness; session 6 – meaning in medicine, body scan, breathing awareness, stretching and body exercises with full consciousness; session 7 – time management, formal practices of mindfulness and compassion; session 8 – personal self-care plan, open consciousness meditation.<br>• Instructor(s): 1 instructor experienced in MBSR.
Sopezki et al., 2020, Brazil	Modified Mindfulness-Based Cognitive Therapy	• Course duration: 8 weeks<br>• Session's duration: 1 weekly session of 2 h<br>• Content: session 1 - Autopilot, mindfulness, primary and secondary experience, raisin technique and body scan; session 2 - being and doing mode, mindfulness paradox, diaphragmatic breathing, mindfulness of breathing; session 3 – body limits and decentering, movements in mindfulness, three-minute practice; session 4 – acceptance, mindfulness in emotional discomfort; session 5 - negativity mental bias, small pleasures, contemplation of the beautiful, mindful walk; session 6 - self-compassion and the three major emotional systems, self-compassion; session 7 - exhaust funnel, connection with others, compassion meditation; session 8 - course review and practices.<br>• Instructor(s): 1 instructor experienced in mindfulness training.
